# A Retrospective Study on the Duration of Stay After Completion and Total Thyroidectomy

**DOI:** 10.7759/cureus.100200

**Published:** 2025-12-27

**Authors:** Olorunleke M Arokoyo, India Rees, Lauren Bolton, Frank Agada

**Affiliations:** 1 Otolaryngology, Surgery Interest Group Africa, Lagos, NGA; 2 Otolaryngology, York and Scarborough Teaching Hospitals NHS Foundation Trust, York, GBR

**Keywords:** completion thyroidectomy, early discharge, inpatient care, thyroidectomy, total thyroidectomy

## Abstract

Introduction

Thyroidectomy is the most common endocrine head and neck surgery. First-time hemithyroidectomies have been shown to have lower rates of complications such as recurrent laryngeal nerve palsy and hypocalcaemia, when compared to total and completion thyroidectomies, due to dissection occurring on one side in hemithyroidectomies. As a result, hemithyroidectomies (low risk) can be performed as day-case surgeries, while higher-risk total and completion thyroidectomies are more likely to have inpatient care postoperatively. This study aims to examine postoperative admission duration and outcomes following completion and total thyroidectomy in a single centre.

Methods

This retrospective, observational, descriptive review included 137 cases of total and completion thyroidectomies carried out at the Department of Otolaryngology, York District Hospital, UK, over four years. The outcome was defined as the presence or absence of complication(s) in the first 30-day postoperative period. Postoperative duration was classified into less than one day, one day to less than two days, two days to less than three days, and three days or more of inpatient care before discharge.

Results

A total of 137 patients met the study criteria. There were 77 total thyroidectomies and 60 completion thyroidectomies. Nineteen patients had a postoperative stay of less than one day, 70 patients had between one day and less than two days postoperative care, 22 patients had two days to less than three days admission, and 26 patients had a postoperative stay of three days or more. There were 104 females and 33 males. The mean age was 51.10 ± 15.42 years, with an age range of 17-90 years. Overall, the most common preoperative diagnosis was cancer (n = 52), all of which had a complete thyroidectomy. Thyrotoxicosis (n = 45) was the second-highest indication.

Hypocalcaemia was the most common cause of delayed discharge postoperatively, accounting for 58.3% (n = 14) of all patients who stayed more than three days after surgery. Of all obese patients, 51% had a postoperative stay of two or more days. A total of 106 patients (80.3%) had a drain inserted, and the use of a drain was related to extended hospital stay postoperatively (Chi-square value = 8.989, p = 0.029). There was no statistically significant relationship between the use of drains and prevention of symptomatic seroma (Chi-square value: 0.011, p = 0.918, Fisher's p = 1.000). There were five (3.6%) cases of postoperative haematoma, and one of those five did not have a drain (Chi-square value = 0.000, p = 0.987, Fisher's p = 1.000). All five postoperative haematoma patients had total thyroidectomy (Chi-square value = 4.044, p = 0.044, Fisher's p *= *0.068). Patients with higher BMI were more likely to have a longer postoperative stay in the hospital (Chi-square value: 24.967, p = 0.003). Of those who stayed two days to less than three days, 86.3% were either overweight or obese, and 84.6% of patients who stayed three or more days were also either overweight or obese.

Conclusion

The use of drains, BMI of the patient, and development of hypocalcaemia are associated with extended in-hospital stay following total and completion thyroidectomy. Planning for early discharge requires careful patient selection, with close monitoring in the observation period before discharge, while ensuring adequate availability of support and proximity to hospital or emergency response systems after discharge, for patient safety.

## Introduction

Thyroidectomy is the most common endocrine head and neck surgery [1]. Although they were historically associated with high morbidity rates, they are now regarded as relatively safe procedures, with good outcomes when performed by high-volume surgeons [2]. Thyroidectomies can be classified based on how much or what part of the gland is removed, for example, a hemithyroidectomy, where one lobe is removed, an isthmusectomy, where the isthmus is removed, or a total thyroidectomy, where the whole gland is removed. Historically, subtotal thyroidectomies were performed, but this is no longer a common practice. They can also be classified as primary or revision operations; revision being where part or what is left of the gland is removed subsequently, as with a completion thyroidectomy.

The indications for total and completion thyroidectomy include management of Graves’ disease, goitre with compressive symptoms and as a treatment option for thyroid malignancies. Known complications include hypocalcaemia, postoperative haemorrhage causing haematoma, infection, seroma, recurrent laryngeal nerve palsy, and superior laryngeal nerve palsy.

Hemithyroidectomies are theoretically expected to have lower rates of complications such as recurrent laryngeal nerve palsy and hypocalcaemia, when compared to total and completion thyroidectomies [3], due to dissection occurring on one side in hemithyroidectomies. Therefore, the recommendation is for hemithyroidectomies (low risk) to be performed as day case surgeries [4], while higher-risk total and completion thyroidectomies are more likely to have inpatient care postoperatively.

Shorter duration of postoperative care has advantages of reduced hospital stay, reduced elective surgery waiting list, comfortable patient experience and financial cost, which is significantly better for both the hospital and the patient [5-8].

This study aims to examine postoperative care duration and outcomes following completion and total thyroidectomy in a single centre. The primary objective is to describe postoperative length of stay distribution after total and completion thyroidectomy and to identify factors associated with extended stay. The secondary objectives are to report the incidence of the following complications: hypocalcaemia, haematoma, recurrent laryngeal nerve palsy, and seroma; to assess whether drains are associated with reduced seroma/haematoma rates and to examine the association of drain use and BMI with length of stay.

## Materials and methods

This is a retrospective, observational, descriptive review of total and completion thyroidectomies carried out at the Department of Otolaryngology, York District Hospital, York, UK, over four years (January 2021 to December 2024). The inclusion criteria are as follows: All patients who had total and completion thyroidectomy via conventional approach under general anaesthesia, for benign or malignant thyroid disease, within the study period. Exclusion criteria included those patients who simultaneously had parathyroidectomy and/or neck dissection.

A total of 137 patients met the criteria, and following retrospective review of their electronic medical records, information collected for each case included age, sex, preoperative diagnosis, type of thyroidectomy, operation time, use of drain, duration of postop stay and outcome. The outcome was defined as the presence or absence of complication(s) in the first 30-day postoperative period [9]. These complications include postoperative haemorrhage causing haematoma, hypocalcaemia, recurrent laryngeal nerve palsy and superior laryngeal nerve palsy, large symptomatic seroma requiring aspiration, and postoperative infection. Hypocalcaemia was managed by following the British Association of Endocrine and Thyroid Surgeons (BAETS) post-thyroidectomy/parathyroidectomy hypocalcaemia guidance, which combines both serum adjusted calcium levels and presence of symptoms for diagnosis, monitoring, and care [10]. Patients with hypocalcaemia were discharged after serum calcium had normalised. 

Postoperative duration was classified into less than one day, one day to less than two days, two days to less than three days, and three or more days of inpatient care before discharge. Short stay was defined as the postoperative period of 23 hours or less before discharge [7,11]. Patients were given safety netting advice on discharge to seek urgent medical care if they had complications before their follow-up appointment. All patients were followed up in a clinic one month after the operation, with further follow-up depending on the histological diagnosis, such as cancer. Patients with persistent vocal cord palsy were seen again six months after surgery. 

Data were collated and analysed using MS Excel (Microsoft Corporation, Redmond, Washington, United States) and IBM SPSS Statistics for Windows, Version 30 (Released 2024; IBM Corp., Armonk, New York, United States), respectively. Categorical variables were compared using the Chi-square test and Fisher’s exact test. Comparison with a p-value less than 0.05 was considered statistically significant.

## Results

A total of 137 patients met the study criteria. There were 77 total thyroidectomies and 60 completion thyroidectomies. Nineteen patients had a postoperative stay of less than one day, 70 patients had between one day and less than two days postoperative care, 22 patients had two days to less than three days postoperative stay, and 26 patients had a postoperative stay of three days or more. 

There were 104 females and 33 males. The mean age was 51.10 ± 15.42 years, with an age range of 17-90 years. The mean operating time for all patients was 95.67 ± 35.23 minutes, while the mean operating time for total thyroidectomy was 109.78 ± 34.72 minutes and 78.47 ± 27.78 minutes for completion thyroidectomy. The mean body mass index (BMI) of all patients was 29.23 ± 6.47 kg/m^2^, with 26.3% having normal BMI, 31.4% overweight, and 40.9% being obese.

**Figure 1:**
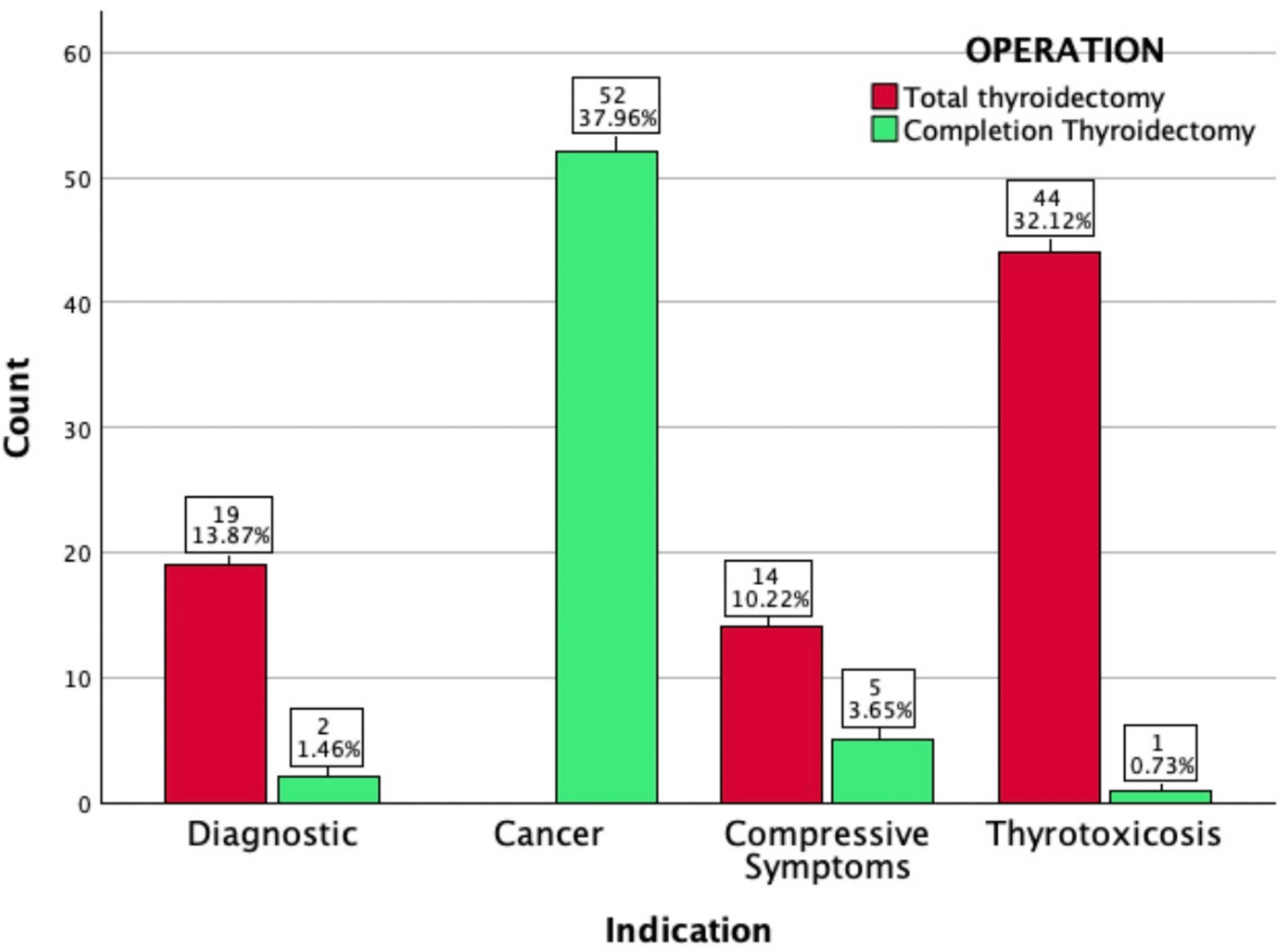
Bar chart showing the type of thyroidectomy performed per preoperative indication Preoperative indications are listed on the X-axis. The red bar represents cases of total thyroidectomy. The green bar represents cases of completion thyroidectomy. The box above each bar states the count of each thyroidectomy type per indication (above), with the percentage of count within the total number of cases in the study (beneath)

Overall, the most common preoperative diagnosis was thyroid cancer (n = 52), all of which had completion thyroidectomy. Thyrotoxicosis (n = 45) was the second-highest indication (Figure 1).

In thyrotoxic patients, 44 of 45 had total thyroidectomy, and one patient with a prior history of thyroid lobectomy had a completion thyroidectomy. All cases of thyroid cancer had completion thyroidectomy following established histological diagnosis. Of 19 patients who had compressive symptoms, 14 had total thyroidectomy, and five had completion thyroidectomy. Harmonic scalpel and bipolar diathermy were utilised as energy sources in 60 cases, while bipolar diathermy was the only energy source in 77 cases. 80.3% (n = 110) of cases had a drain inserted. 

Overall complications included 24 patients with hypocalcaemia (17.5%), nine patients with transient vocal cord palsy (three with permanent vocal cord palsy (2.2%)). Five patients (3.6%) had postoperative haematoma (Tables 1-2).

**Table 1:** Complication per preoperative indication This table shows the count of postoperative complications (row captions) for each preoperative indication (column caption). Chi-square test was performed for tabulated variables; p-value < 0.05 was considered statistically significant

Postoperative complication		Preoperative indication						
			Diagnostic thyroidectomy	Cancer	Compressive symptoms	Thyrotoxicosis	Chi-square value	p-value
	Infection		2	5	3	7	1.144	0.766
	Seroma		0	4	2	10	8.452	0.038
	Hypocalcaemia		2	8	5	9	2.302	0.512
	Vocal cord palsy		2	2	1	7	4.477	0.214
	Haematoma		2	0	0	3	5.915	0.116

**Table 2:** Count of complications per operation This table shows the frequency of postoperative complications (row captions) with each type of thyroidectomy performed (column caption). Chi-square test and Fisher's exact test were performed for tabulated variables; Fisher's p-value <0.05 was considered statistically significant

	Thyroidectomy				
Postoperative complications		Total thyroidectomy	Completion thyroidectomy	Chi-square value (p-value)	Fisher's exact test p-value
	Infection	11	6	0.570 (0.450)	0.603
	Seroma	12	4	2.600 (0.107)	0.179
	Hypocalcaemia	15	9	0.469 (0.494)	0.651
	Vocal cord palsy	8	4	0.585 (0.444)	0.550
	Haematoma	5	0	4.044 (0.044)	0.068

Hypocalcaemia was the most common cause of delayed discharge postoperatively (Chi-square value = 30.943, p < 0.001), accounting for 58.3% (n = 14) of all patients who stayed more than three days after surgery. Fifty-one percent of all obese patients had a postoperative stay of two or more days (Chi-square value = 24.967, p = 0.003).

## Discussion

Improvement in thyroidectomy outcomes over time, coupled with cost-efficiency and patient convenience, has influenced a growing trend towards shorter postoperative hospital stay [12]. While some low risk thyroid operations (such as diagnostic lobectomies) are recommended to be performed as day cases [8], total and completion thyroidectomies are more likely to be done as inpatient operations, due to the potential complication rates and severity associated with the preoperative diagnosis, relatively larger extent of the surgery and theorised difficulty expected with a revision thyroidectomy, among other factors [13].

The most serious complications are haemorrhage with an endangered airway, bilateral recurrent laryngeal nerve palsy with airway compromise, and severe hypocalcaemia. These are more likely to occur in the first six hours postoperatively, with a smaller percentage occurring outside this window but within the first 24 hours [11].

Post-thyroidectomy hypocalcaemia is a common complication [7], theorised to result from injury to the parathyroid gland or vessels. The type of surgery and indication have also been suggested as risk factors. The incidence of hypocalcaemia has been shown to increase from the first six hours to the first postoperative day, where it peaks [14]. In our study, hypocalcaemia was the most common complication, detected on checking serum calcium at six-hour, 12-hour, and/or 24-hour postoperative intervals, with all cases subsequently managed as inpatients till calcium level was safe for discharge. The majority (n = 22) of all 24 hypocalcaemic patients had mild adjusted serum levels, while two of 24 patients had severe hypocalcaemia (both had total thyroidectomy, one for thyrotoxicosis, the other for compressive symptoms). 

To reduce the severity and readmission rates from temporary hypocalcaemia, some reports have demonstrated with success [15] the use of prophylactic calcium and vitamin D, with regular monitoring to confirm that hypocalcaemia is indeed transient [16]. 

One hundred and six patients (80.3%) had a drain inserted and removed when output was less than 40 mL in 24 hours. The use of a drain was associated with extended hospital stay postoperatively (Chi-square value: 8.989, p = 0.029). There was no statistically significant relationship between the use of drains and prevention of symptomatic seroma (Chi-square value: 0.011, p = 0.918, Fisher's p = 1.000), as 11.8% (n = 13) of patients who had drains developed seroma, and 11.1% (n = 3) who had no drains also developed seroma. There were five (3.6%) cases of postoperative haematoma, and one of those five did not have a drain (Chi-square value = 0.000, p = 0.987, Fisher's p = 1.000). All five postoperative haematoma patients had total thyroidectomy (Chi-square value = 4.044, p = 0.044, Fisher's p = 0.068), which, although not statistically significant in this study, has been established as a significant risk factor for bleeding [17]. Three of these patients had total thyroidectomy for thyrotoxicosis.

While safety, with the aim of preventing a compressive haematoma, informs the decision to put a drain in place, its use could depend on factors including indications, type of operation, intraoperative findings, and level of experience of the surgeon [11]. Adequate home support, proximity to the hospital, and other criteria for postdischarge care must be ensured regardless. 

Vocal cord palsy causing airway compromise (usually bilateral) is rare [7]. Unilateral recurrent laryngeal palsy causing voice hoarseness is usually due to neuropraxia and is expected to resolve in most cases, and as such is not an indication for admission. Total thyroidectomy, reoperation, and retrosternal goitre are known to increase the risk of recurrent laryngeal nerve palsy, and total thyroidectomy significantly increases the risk of permanent nerve palsy [17]. A total of 6.6% (n = 9) had temporary recurrent laryngeal nerve palsy, and 2.2% (n = 3) of those had permanent palsy. Eight patients had palsy after total thyroidectomy, and four had palsy after completion thyroidectomy (Chi-square value: 0.585, p = 0.444, Fisher's p = 0.550).

Patients with higher BMI were more likely to have a longer postoperative stay in the hospital (Chi-square value: 24.967, p = 0.003). Moreover, 86.3% of those who stayed two days to less than three days were either overweight or obese, and 84.6% of patients who stayed three or more days were also either overweight or obese. This may be useful in preoperative counselling and consenting of patients.

The limitations of this study include retrospectively collated data from a single institution, which restricts its generalisability beyond the study population. The design and methodology also open the study up to selection and information bias. Relatively small sample size, with lower statistical power, limits the representation of the results within the general population and their reliability. To establish causation and increase generalisability, a prospective, larger sample size, multicentre study will be better suited, with multivariate analysis to account for confounding factors.

## Conclusions

The use of a drain, BMI of the patient, and development of hypocalcaemia are associated with extended in-hospital stay following total and completion thyroidectomy. While this study shows a link between these factors and extended hospital stay, a larger sample size, multicentre study, with multivariate analysis is needed to help establish causation. Given the benefits of early discharge to the patient and the hospital, safe measures to reduce these factors, if established, can be useful for service improvement. These measures could include preoperative counselling and planning for patients with high BMI, consideration of prophylactic calcium and vitamin D with regular monitoring to reduce the severity of hypocalcaemia, and the decision to use a drain made based on factors including the indication, type of thyroidectomy, and intra-operative findings. Planning for early discharge requires careful patient selection, with close monitoring in the observation period before discharge, while ensuring adequate availability of support and proximity to hospital or emergency response systems after discharge, for patient safety. 
